# Thoraco‐Omphalopagus Conjoined Twins Following IVF With Day 5 Embryo Transfer: A Rare Case Report and Multidisciplinary Management Approach

**DOI:** 10.1002/ccr3.71288

**Published:** 2025-12-17

**Authors:** Patryk Piekos, Natalia Ravelo, Rodrigo Ruano, George Attia

**Affiliations:** ^1^ Reproductive Endocrinology and Infertility Division, Department of Obstetrics, Gynecology and Reproductive Sciences University of Miami, Miller School of Medicine Miami Florida USA; ^2^ Maternal Fetal Medicine, Department of Obstetrics, Gynecology and Reproductive Sciences University of Miami, Miller School of Medicine Miami Florida USA

**Keywords:** assisted reproductive technology (ART), conjoined twins, day 5 embryo transfer, in vitro fertilization (IVF), intracytoplasmic sperm injection (ICSI), thoraco‐omphalopagus

## Abstract

Conjoined twinning is a rare and severe complication of monozygotic pregnancies. Assisted reproductive technologies (ART), including in vitro fertilization (IVF), may increase the risk of such anomalies. We report a case of thoraco‐omphalopagus conjoined twins following a day 5 embryo transfer after IVF with intracytoplasmic sperm injection (ICSI). The diagnosis was made by ultrasound at 8 weeks' gestation and confirmed the presence of fused thoracic and abdominal structures with a shared heart. The pregnancy was managed through multidisciplinary counseling, and termination was performed following spontaneous fetal demise. This case underscores the importance of early imaging, detailed embryologic understanding, and coordinated care in managing rare IVF‐associated anomalies.

**Summary**

- Thoraco‐omphalopagus conjoined twins are a rare and severe anomaly, often with a fatal prognosis when cardiac structures are shared.
- This case, following IVF, highlights the importance of early diagnosis, multidisciplinary management, and comprehensive patient counseling in complex reproductive decisions.
- Assisted reproductive technology may increase the risk of monozygotic twinning.

## 1 Introduction

Conjoined twins are an extremely rare phenomenon, with an estimated global prevalence of approximately 1 in 50,000 to 1 in 200,000 live births, accounting for about 0.5% of monozygotic twin pregnancies [1]. The literature suggests that assisted reproductive technology (ART) might increase the incidence of conjoined twins two and a half to tenfold, potentially due to mechanical manipulation of the zona pellucida during ICSI, assisted hatching, or extended in vitro culture, all of which may interfere with normal blastomere cleavage or embryonic polarity [2].

Conjoined twins arise from delayed cleavage of a fertilized egg beyond 13 days post‐fertilization, resulting in incomplete embryonic separation [3]. Thoraco‐omphalopagus twins, sharing thoracic and abdominal structures, represent a severe form with poor prognosis, particularly when cardiac structures are shared [4]. Thoraco‐omphalopagus twins account for 28%–32% of all conjoined twin cases, making them one of the most common forms of this anomaly [5].

This case report details a thoraco‐omphalopagus conjoined twin pregnancy following in vitro fertilization (IVF) with fresh transfer of three blastocysts that underwent intracytoplasmic sperm injection (ICSI) and its subsequent management. The report further explores embryologic and clinical factors, including the use of a 0PN embryo (an embryo with no visible pronuclei, often assumed abnormal but occasionally viable), and emphasizes the role of multidisciplinary coordination in navigating diagnosis and reproductive counseling.

## 2 Case History/Examination

A 42‐year‐old G1P0 woman presented for IVF treatment due to primary infertility associated with advanced maternal age (AMA). The patient had no history of smoking, alcohol, or drug use, and no family history of congenital abnormalities. Her menstrual cycles were regular, and ovarian reserve testing revealed an anti‐Müllerian hormone level of 1.43 ng/mL with an antral follicle count of 10. She had previously undergone one cycle of letrozole with intrauterine insemination without success. The partner, a 39‐year‐old male, had no significant medical or family history. The couple was non‐consanguineous.

The patient underwent two IVF cycles. The first cycle involved stimulation with Gonal‐F 300 IU recombinant follicle‐stimulating hormone (FSH, 300 IU) and human menopausal gonadotropin (hMG, 75 IU) for eight days, with a gonadotropin‐releasing hormone (GnRH) antagonist, initiated on day 5, and an hCG trigger (5000 IU) 34 h before oocyte retrieval. Four mature oocytes were retrieved, and all underwent ICSI with fresh sperm from her partner. Two fresh grade 8AP embryos without assisted hatching were transferred on Day 3, but the pregnancy test was negative 12 days later.

The second IVF cycle included recombinant human growth hormone (somatropin, 1.6 mg daily) starting three days before stimulation and continued through the day of trigger. Ovarian stimulation was achieved using recombinant FSH (225 IU) and hMG (150 IU) for eight days, with a GnRH antagonist initiated on Day 6. The hCG trigger (5000 IU) was administered 34 h before oocyte retrieval. Four mature oocytes were retrieved, and all underwent ICSI. Progesterone supplementation was initiated for luteal support in preparation for a fresh transfer.

Normal fertilization is typically confirmed on Day 1 after insemination by the presence of two pronuclei (2PN), representing the separate genetic contributions from the oocyte and sperm. This 2PN stage is considered the gold standard indicator of successful fertilization in IVF. In contrast, embryos with no visible pronuclei (0PN) may arise from abnormal fertilization or from early or asynchronous pronuclear fading, and while often presumed non‐viable, some 0PN embryos can develop into blastocysts and result in healthy pregnancies [6]. Embryo quality is further assessed using the Society for Assisted Reproductive Technology (SART) grading system, which evaluates blastocysts based on the degree of expansion and the morphology of the inner cell mass and trophectoderm to estimate implantation potential.

On Day 1 post‐retrieval, three embryos showed two visible pronuclei (2PN), indicating normal fertilization, and one was 0PN. On Day 5, the embryo from the 0PN group became an expanded blastocyst graded as good/fair by the Society for Assisted SART grading system and was selected for transfer. Two additional embryos from the 2PN group were also included in the transfer. Thus, all embryos created in this cycle were ultimately included in the fresh Day 5 transfer.

**FIGURE 1:**
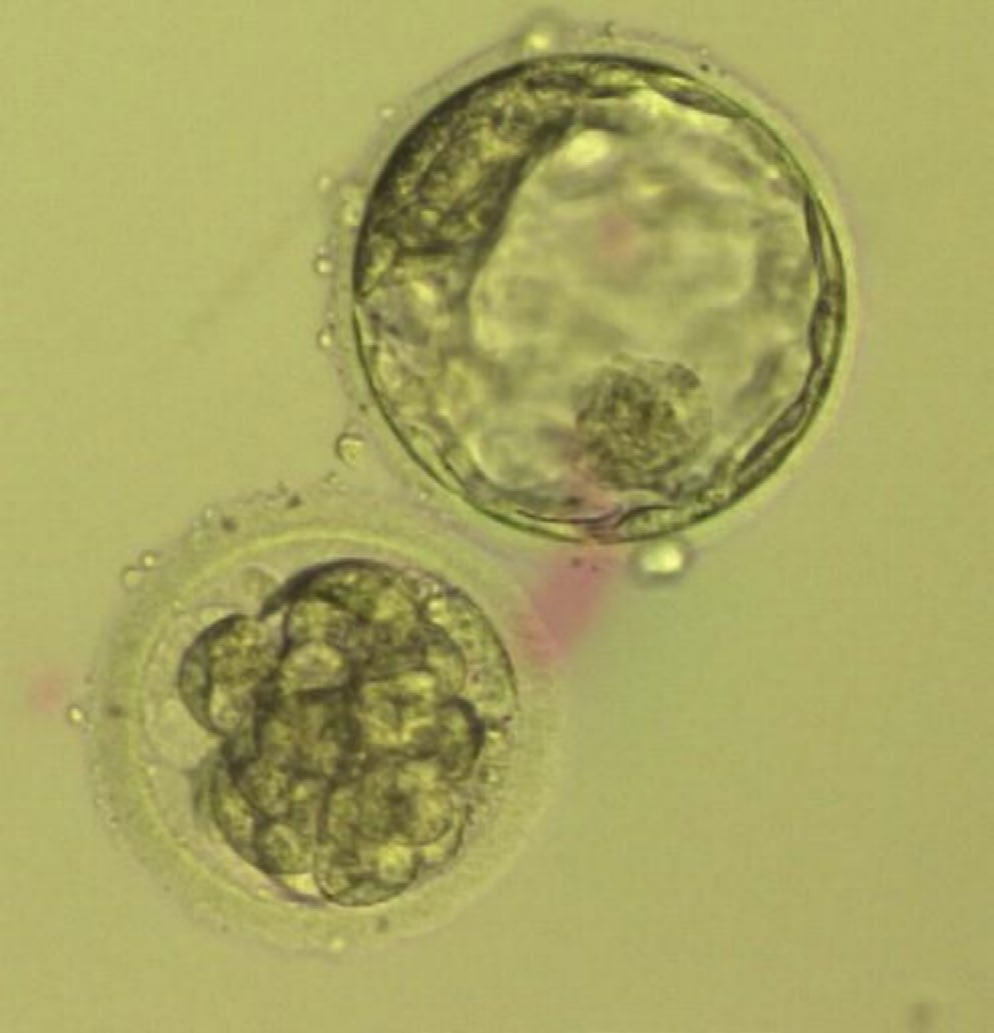
Transferred Day 5 2PN expanded blastocyst with poor/fair morphology (top) and a 2PN early blastocyst (bottom).

**FIGURE 2:**
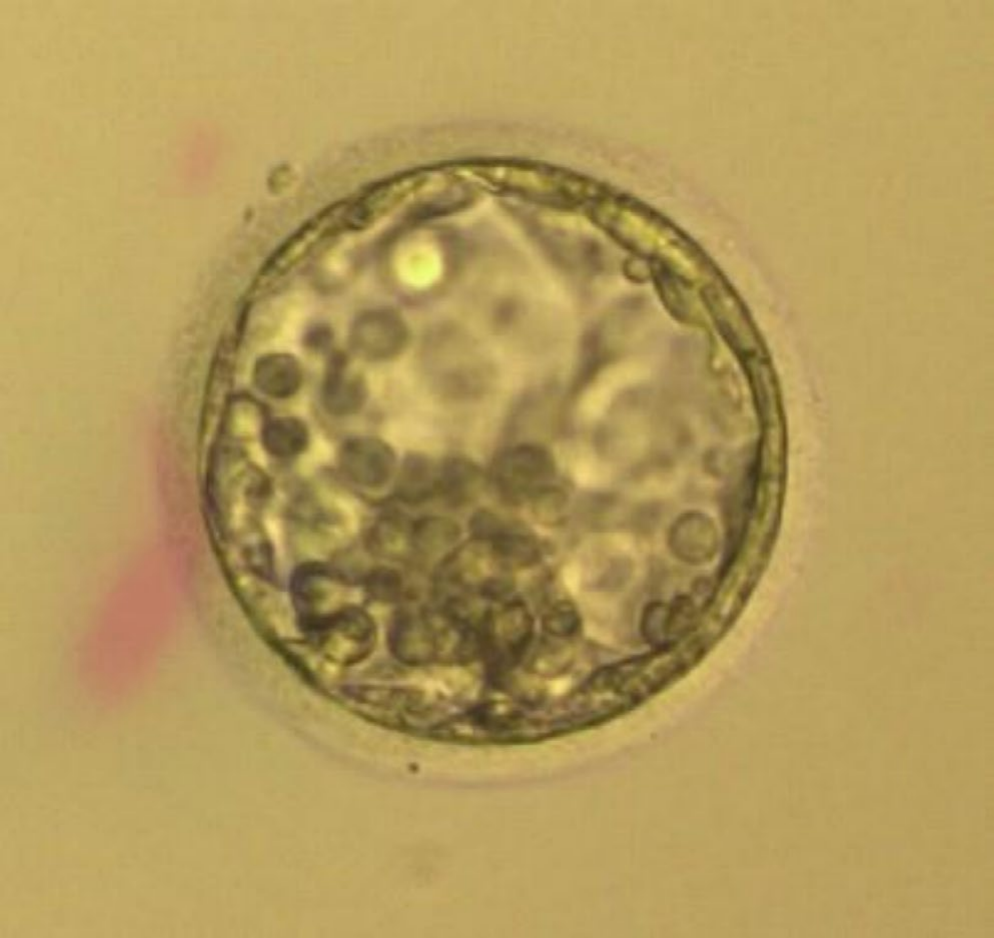
Transferred Day 5 0PN expanded blastocyst graded as good/fair.

In summary, three fresh embryos were transferred on Day 5: a 2PN early blastocyst, a 2PN expanded blastocyst with poor/fair morphology (Figure 1), and a 0PN expanded blastocyst graded as good/fair (Figure 2). None of the embryos underwent assisted hatching. The decision to transfer the 0PN embryo was made after detailed counseling and due to limited embryo yield. While typically not prioritized, emerging evidence and clinical experience suggest that some 0PN embryos may undergo normal development and implantation [6].

Prior to transfer, the patient was counseled on the risk of multiple pregnancy given her history of AMA, previously failed IVF, and low ovarian reserve. A positive pregnancy was achieved.

At the first ultrasound, a single intrauterine gestational sac with cardiac activity was identified, with a crown‐rump length (CRL) of 5.42 mm corresponding to 6 weeks and 2 days' gestation, consistent with the transfer date. A small subchorionic hemorrhage measuring 1.17 cm × 0.95 cm was noted.

At a follow‐up ultrasound at 8 weeks, two fetuses were identified, confirming a monochorionic‐monoamniotic twin gestation with thoracic and abdominal fusion and a shared cardiac structure. Fetus A had a CRL of 19.92 mm, and Fetus B measured 18.43 mm, both consistent with the embryo transfer date. Cardiac activity was shared between the fetuses. The patient was referred to maternal‐fetal medicine (MFM) for further management.

**FIGURE 3:**
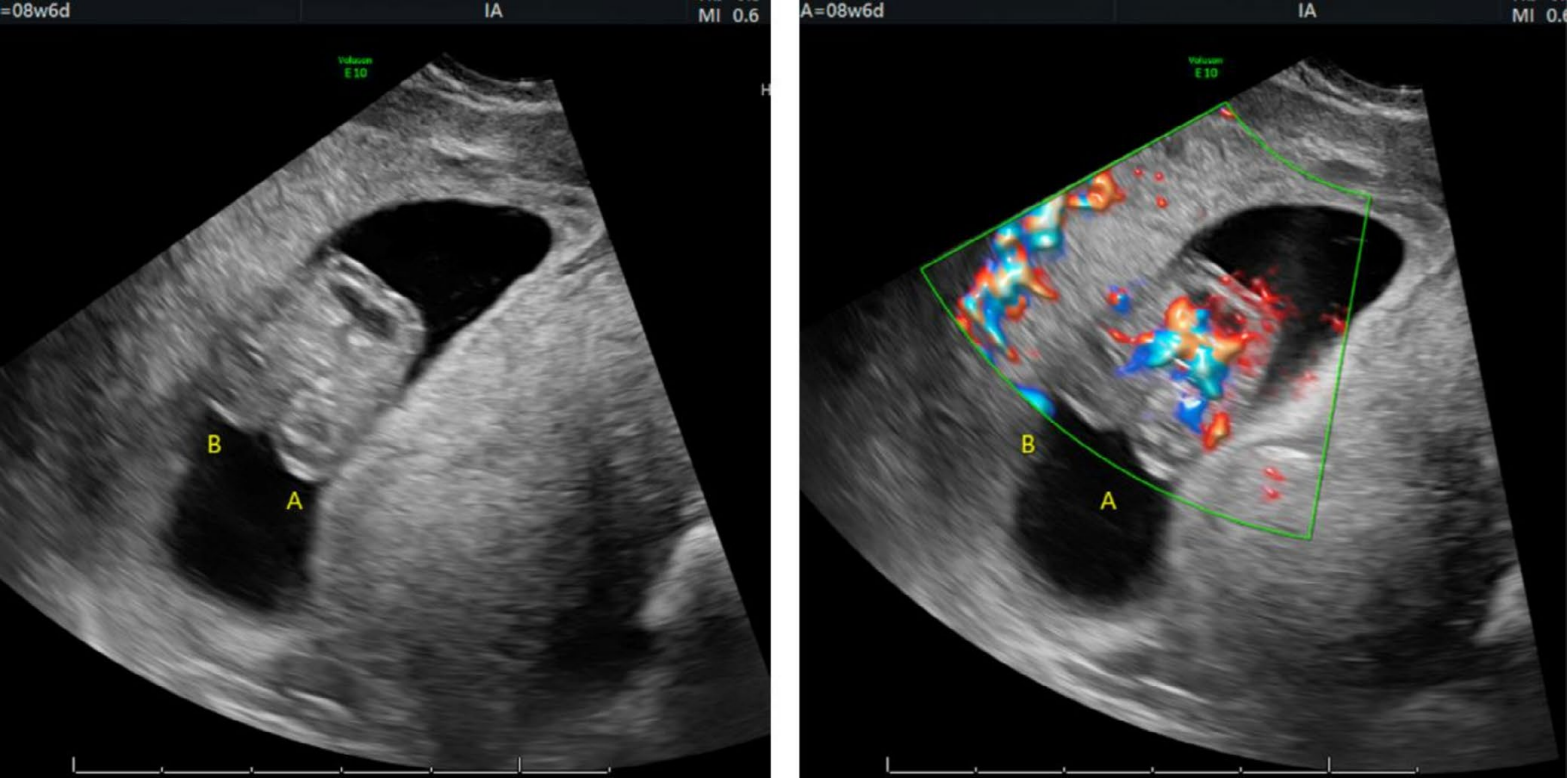
Thoraco‐omphalopagus conjoined twins at 8 weeks and 6 days with Doppler color flow demonstrating a shared heart.

**FIGURE 4:**
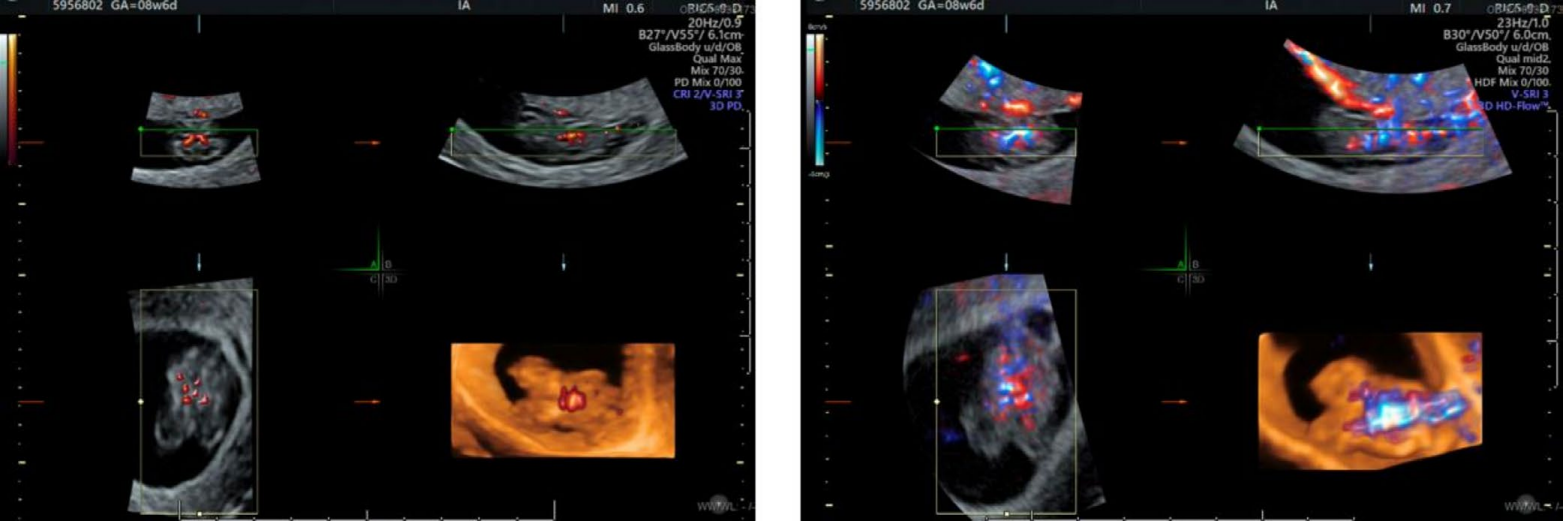
3D ultrasonography of thoraco‐omphalopagus conjoined twins at 8 weeks and 6 days.

At 8 weeks and 6 days, a follow‐up ultrasound by MFM reconfirmed thoraco‐omphalopagus conjoined twins with one heart (Figures 3 and 4). No other anatomical anomalies were identified.

## 3 Differential Diagnosis

Although monochorionic monoamniotic twins share a single amniotic sac, they maintain distinct body contours and organ systems. In this case, the demonstration of continuous tissue planes and a shared cardiac structure on both 2D and 3D imaging confirms true conjoining rather than mere proximity of two separate fetuses.

The twin reversed arterial perfusion (TRAP) sequence, which involves an acardiac twin receiving circulation from a pump twin and can sometimes be misinterpreted as conjoining on early ultrasound, was effectively excluded by the presence of a single, structurally shared heart and the absence of a separate acardiac mass.

Additionally, while a fused placental mass or closely positioned fetuses within the uterus may simulate the appearance of conjoined twins, detailed anatomical assessment—particularly using Doppler studies and 3D ultrasonography—confirms true tissue continuity and shared vital organs.

## 4 Conclusion and Results

A multidisciplinary team—including specialists in reproductive endocrinology, maternal‐fetal medicine, and complex family planning—managed the patient. Counseling emphasized the lethality of the condition due to the shared cardiac structure [1]. Options for expectant management, continuation of pregnancy, or termination were discussed. The patient opted for suction dilation and curettage (D&C) after informed consent and was instructed to stop progesterone supplementation.

Before the patient's procedure, a follow‐up ultrasound at 10 weeks and 2 days showed the absence of fetal cardiac activity. Crown‐rump lengths measured 9 weeks and 1 day and 9 weeks and 4 days. Subsequently, the patient experienced an incomplete miscarriage, and the D&C was performed without complications. Pathological examination confirmed products of conception, which were described as 9 cm of gestational villi and decidua. However, there were no discernible embryonic or fetal parts.

## 5 Discussion

This case highlights the challenges of managing conjoined twins, particularly those with severe anomalies such as shared cardiac structures. While conjoined twins are rare, their incidence may be increased in the context of ART, possibly due to the manipulation of the zona pellucida [2, 4]. Proposed mechanisms include altered blastomere orientation, abnormal cell adhesion due to extended in vitro culture, and micromanipulation of embryos during ICSI or assisted hatching—any of which may interfere with the natural timing and symmetry of embryonic cleavage [2].

The prenatal diagnosis of thoraco‐omphalopagus twins underscores the importance of early multidisciplinary evaluation involving reproductive endocrinology, maternal‐fetal medicine, and complex family planning [7]. Despite advances in imaging and surgical separation techniques, the outcomes for twins sharing vital organs, such as the heart, remain poor [2]. The degree of cardiac involvement is the main determinant of prognosis [5]. Furthermore, symmetric twins can be more challenging to manage compared to asymmetric (heteropagus or “parasitic”) twins due to the need to balance ethical and medical decisions affecting both non‐parasitic twins.

Studies have shown that approximately 70%–75% of conjoined twins are female, despite the fact that monozygotic twinning itself is not sex‐biased. This skewed sex ratio is thought to result from a higher rate of in utero loss among male conjoined twins, suggesting that male fetuses with conjoining may have a lower survival rate than their female counterparts [8]. In our case, fetal sex could not be determined due to the lack of identifiable fetal anatomy on pathological examination.

Grøndahl et al. [9] used time‐lapse imaging to track embryogenesis from zygote to blastocyst formation, revealing cleavage irregularities in embryos that later resulted in conjoined twins. Such technology could serve as a valuable tool for identifying embryos at higher risk of developmental abnormalities. However, this tool is not yet routinely used in clinical IVF settings and cannot currently prevent conjoining. In our case, standard fertilization assessment was performed. One of the embryos transferred was a 0PN embryo, which is often presumed to be abnormal due to the absence of visible pronuclei. Nonetheless, some 0PN embryos may arise from asynchronous pronuclear fading and can develop into viable blastocysts [6].

There are only 18 published cases of conjoined twins following ART. Of these, only three involved the transfer of Day 5 embryos. In one case, two fresh embryos were transferred on Day 5 [2]; in another, two fresh Day 3 embryos and one fresh Day 5 embryo were transferred [4]. The third case, reported by [9] involved a Day 5 transfer after a frozen embryo transfer cycle. This report represents the fourth known case of conjoined twins following a Day 5 embryo transfer.

In our case, an 18‐h check after insemination revealed three 2PN embryos and one 0PN embryo. Grøndahl et al. [9] reported that in their case, at 19 h post‐insemination, 2PN were observed, with one pronucleus fading before the other—a 2‐h asynchrony that may reflect abnormal zygotic development. Although we cannot definitively determine which embryo resulted in the pregnancy, we hypothesize that the more morphologically advanced 0PN‐derived blastocyst was responsible. Its developmental potential underscores the need for nuanced consideration of 0PN embryos in low‐yield IVF cycles.

Preimplantation genetic testing (PGT) was not performed in this case due to the low number of embryos and financial constraints. While PGT may assist in detecting chromosomal abnormalities, it does not screen for morphogenetic events such as conjoining. Future integration of genomic, morphokinetic, and molecular markers may improve risk prediction in such rare outcomes.

This case also illustrates the value of a multidisciplinary care model. The reproductive endocrinology team played a critical role in embryo selection, transfer decision‐making, and early pregnancy monitoring. The maternal‐fetal medicine team contributed through detailed anatomical diagnosis, risk stratification, and perinatal counseling. Finally, the family planning team provided comprehensive support, including ethical consultation and procedural management. The coordinated involvement of these specialties was essential in guiding the patient through a complex and emotionally challenging clinical scenario.

By presenting one of the few reported cases of thoraco‐omphalopagus conjoined twins following Day 5 transfer, our report contributes new clinical insights and highlights opportunities for future research into embryo behavior, ART safety, and personalized patient counseling.

## 6 Conclusion

Thoraco‐omphalopagus conjoined twins represent a rare and severe anomaly with significant implications for prognosis and clinical management. This case—occurring after IVF with Day 5 blastocyst transfer and inclusion of a 0PN embryo—illustrates both the potential complexity of embryo selection and the importance of early, high‐resolution imaging for diagnosis.

Early multidisciplinary involvement was critical in guiding the patient through complex reproductive decisions, including counseling on lethality, pregnancy continuation, and timing of termination. This case underscores the importance of clear communication, ethical sensitivity, and coordinated care in ART‐related anomalies.

Future research should explore the utility of time‐lapse imaging in identifying abnormal cleavage patterns and investigate whether molecular or genetic markers can help predict the risk of developmental anomalies like conjoining. Additionally, clearer clinical guidelines regarding the use of 0PN embryos, particularly in patients with low embryo yield, may improve patient safety and informed consent.

## Author Contributions

**Patryk Piekos:** writing – original draft. **Natalia Ravelo:** formal analysis. **Rodrigo Ruano:** conceptualization. **George Attia:** conceptualization, formal analysis.

## Consent

Written patient consent has been signed and collected in accordance with the journal's patient consent policy.

## Conflicts of Interest

The authors declare no conflicts of interest.

## Data Availability

Data sharing is not applicable to this article as no new data were created or analyzed in this study.
